# Human adenovirus species C recombinant virus continuously circulated in China

**DOI:** 10.1038/s41598-019-46228-2

**Published:** 2019-07-05

**Authors:** Jianfang Yang, Naiying Mao, Chuangye Zhang, Binzhi Ren, Hong Li, Na Li, Jing Chen, Ruifu Zhang, Hong Li, Zhen Zhu, Wenbo Xu

**Affiliations:** 1 Shanxi Provincial Center for Disease Control and Prevention, Taiyuan, 030012 People’s Republic of China; 2 NHC Key Laboratory of Medical Virology and Viral Diseases, National Institute for Viral Disease Control and Prevention, Chinese Center for Disease Control and Prevention, No.155, Changbai Road, Changping District, Beijing, 102206 People’s Republic of China; 3 Medicine College, Anhui University of Science and Technology, Huainan, 232001 People’s Republic of China

**Keywords:** Viral epidemiology, Viral infection

## Abstract

To date, at least three lineages (Lineage 1–3) that are related to recombinant human adenovirus species C (HAdV-C) have been identified in China. Among them, Lineage 1 includes two Chinese strains, strain KR699642-CHN-20093 (CBJ11) and strain MF315029-CHN-2013 (BJ09), which were collected in Beijing in 2009 and 2013, respectively. Herein, we performed genomic and bioinformatics analysis of two HAdV-C strains (strain SX-2000-140 and strain SX-2004-327) that were isolated from the feces of two healthy children in Shanxi province of China in 2000 and 2004, respectively. Results revealed that the genomes of both Shanxi strains had the highest homology to two Chinese HAdV-C strains belonging to Lineage 1 and harbored the genetic elements of these two strains, thereby presuming that Lineage1 has been circulated in mainland of China for decades. In addition, though the viruses in Lineage 1 showed slightly different recombinant patterns resulting from the recombinant events among the five types of HAdV-C, all the Lineage 1 viruses shared the highest sequence similarities with the HAdV-2 prototype strain (NC_001405-USA-1953) across the genome, especially in the major capsid genes including hexon, and fiber. These results indicated that Lineage 1 viruses that were associated with recombinants shared a common ancestor that is closely related to the HAdV-2 virus. Our current findings confirmed that frequent recombination among the different HAdV-C types might be an important driving force for the molecular evolution of HAdV-C. Therefore, there is a strong need for further comprehensive and systematic monitoring, detection, and research on HAdV-C.

## Introduction

Human adenoviruses (HAdVs) are non-enveloped, icosahedral, double-stranded DNA viruses that belong to the family *Adenoviridae* and genus *Mastadenovirus*. The size of the HAdV genome is about 36 kb^1,2^. The primary adenovirus antigens are three viral capsid proteins, including the hexon, penton base, and fiber^3^. Based on the results of neutralizing and hemagglutination assays, genome sequencing and functional characterization, and analysis of phylogenetic and biological characteristics, HAdVs can be divided into seven species (A-G) with more than 90 types that have been reported (http://www.hadvwg.gmu.edu). Homologous recombination between different types of HAdVs is a major driving force for the molecular evolution of HAdVs and leads to the generation of novel emerging pathogens^4^. However, the mechanisms underlying the viral recombination remain unclear.

HAdVs are highly contagious pathogens that are known to be involved in a broad spectrum of human diseases, including respiratory diseases^5^, conjunctivitis, cystitis^6^, encephalitis^7^, and gastroenteritis^8^. Species C members are recognized as the primary pathogens responsible for respiratory tract infections among pediatric patients^9–11^, especially infants who are less than two years old^12^. Generally, HAdV-C infection may be asymptomatic or mild and self-limiting but could lead to severe effects in immunocompromised hosts, such as transplant recipients^13–15^. Given that HAdV-C is capable of causing persistent infections in intestinal T lymphocytes of the digestive tract, the virus can remain in feces for months and even years because of intermittent excretion, even though the primary infection can be respiratory^16^.

So far, six HAdV types of species C, namely, HAdV-1, HAdV-2, HAdV-5, HAdV-6, HAdV-57, and HAdV-89 have been formally recognized. Of these, The first HAdV-57 isolated from the feces of a healthy child in 2001^17^, and the first HAdV-89 identified from the feces of an immunosuppressed patient in 2015^18^, both were identified as recombinant viruses. The fiber gene of HAdV-57 was found to be similar to that of HAdV-6, and HAdV-57 was found to harbor a unique hexon distinguished by its loop-2 motif^17^; While HAdV-89 had a novel penton base sequence^18^. In addition, recent studies identified a recombinant HAdV-C strain whose gene regions mainly originated from HAdV-1 and HAdV-2, in Beijing, China^19,20^. To determine the prevalence of this recombinant HAdV-C strain in China, a retrospective study was conducted. Herein, we performed genomic and bioinformatics analyses of two HAdV-C strains (strain SX-2000-140 and strain SX-2004-327) that were isolated from the feces of healthy children in the Shanxi province of China in 2000 and 2004 during the acute flaccid paralysis (AFP) surveillance program of the national poliovirus surveillance. The two target strains showed high homology with that of a previously reported recombinant HAdV-C strain.

## Materials and Methods

### Ethical statement

The present study was approved by Ethics Review Committee of Shanxi provincial Center for Disease Control and Prevention. All methods were performed in accordance with the relevant guidelines and regulations. Written informed consents were obtained from legal guardians for the collection of stool specimens from two healthy children less than 15 years of age for pathogenic identification as part of the AFP surveillance program.

### Virus amplification and DNA extraction

The complete procedures for the collection, processing, and virus isolation of the stool samples were performed according to World Health Organization (WHO) standard protocol^21^. Both target viruses underwent three passages in human rhabdomyosarcoma (RD) cells to obtain high-titer stocks before use in subsequent experiments. Viral nucleic acids were extracted from the cultured virus suspensions using a QIAamp DNA mini kit (Qiagen, Valencia, CA, USA) following the manufacturer’s instructions.

### Full-length genome sequencing and annotation

Eight overlapping polymerase chain reaction (PCR) fragments covering the entire genome were amplified using the Platinum PCR SuperMix (Invitrogen, Carlsbad, CA, USA) following previously described protocols^20^. Primers for specific HAdV-C PCR amplification were synthesized as previously reported^20^. Following the PCR amplification of the full-length genome, the amplified DNA was used as template for sequencing using Sanger chemistry using the BigDye Terminator v3.1 Cycle Sequencing Kit (Thermo Fisher Scientific, Waltham, MA, USA). Sequence ladders were generated on the ABI Prism 3100 Genetic Analyzer (Life Technologies, Japan). Sequences were assembled and edited using Sequencher 5.0 (Genecodes Corp., Ann Arbor, MI, USA). To obtain high-quality data, we used a minimum threefold coverage for both directions across the genomes. In addition, any questionable sites identified during the sequence assembly and genome annotation were re-sequenced to clarify the ambiguities. Genome annotation was performed using Artemis software version 16.0.0 (Sanger, UK) and HAdV-2 prototype strain (NC_001405) was used as the template for the genomic comparative analysis.

### Bioinformatics analysis

Multiple sequence alignment was performed using MAFFT software version 7.311 (http://mafft.cbrc.jp/alignment/software/). Phylogenetic trees were constructed using MEGA software version 6.0 by the neighbor-joining and maximum likelihood methods, respectively^22^. The phylogenetic tree inference was tested with the bootstrap method with 1000 replications, and the bootstrap values greater than 80% were indicated in the tree and recognized as strong support. BioEdit software version 7.0.4.1 (http://www.mbio.ncsu.edu/bioedit/bioedit.html) was used to generate the similarity between the sequences of different genes across the genomes. The phylogenetic network constructed to investigate the evolutionary histories of the strains based on the whole-genome sequences (WGSs) was generated using SplitsTree4 software version 4.14.6 with default parameters (http://www.splitstree.org/). To identify the potential recombinant events, bootscanning analyses were performed using SimPlot software version 3.5.1 (https://sray.med.som.jhmi.edu/SCRoftware/simplot/). Parameters were set to the default settings as follows: window size of 5000 bp, step size of 100 bp, gap stripping, 100 replicates, kimura (2-parameter), and neighbor-joining.

### Dataset and nucleotide sequence accession numbers

For genome analysis, a total of 27 HAdV-C WGSs generated from 1953 to 2013 from seven countries were directly downloaded from the GenBank database. All the sequences listed were indicated by the GenBank accession number, followed by the country of origin and the year of sample collection. Two Shanxi strains in this study were submitted to GenBank with the accession numbers MK165452 and MK165453. Among the 27 sequences, six belong to the HAdV-C prototype strains, namely, HAdV-1 (AF534906-USA-1953), HAdV-2 (NC_001405-USA-1953), HAdV-5 (AC_000008-USA-1953), HAdV-6 (FJ349096-USA-1953), HAdV-57 (HQ003817-RUS-2001), and HAdV-89 (MH121097-DEU-2015); four were collected from China from 2009 to 2013 (KR699642-CHN-2009, MF315028-CHN-2012, MF315029-CHN-2013, and KF951595-CHN-2013); and 17 were collected from four countries from 1987 to 2008 (LC068713-JPN-1987, KF268310-USA-1992, LC068714-JPN-1993, JX173078-ARG-2000, JX173080-EGY-2001, JX173081-EGY-2001, JX173079-ARG-2002, KX384959-USA-2002, LC068716-JPN-2003, JX173082-USA-2003, JX173083-USA-2003, JX173084-USA-2003, LC068717-JPN-2004, LC068718-JPN-2004, KF268129-USA-2005, JX423389-USA-2007, and KF268199-USA-2008) (Supplementary Table S1).

## Results

### Genomic characterization and comparative genomic analysis

To investigate the genomic characteristics of the two target Shanxi HAdV strains, their WGSs were sequenced and analyzed. Similar to the genome length of the HAdV-2 prototype strain (NC_001405, 35,937 bp), the genome sizes of strains SX-2000-140 and SX-2004-327 were determined to be 35,949 bp and 35,932 bp, respectively, with corresponding GC contents of 55.25% and 55.20%. Coding annotations of the genomes of strains SX-2000-140 and SX-2004-327 and the corresponding genome locations are listed in Table 1. A total of 38 putative coding regions for both Shanxi HAdV strains were identified and organized in a similar manner as the genomes of other viruses within HAdV-C.

**Table 1:** Genome coding annotations of strains SX2000-140 and SX2004-327.

Gene	Product	Coding annotation	
		SX-2004-327	SX-2000-140
E1A	Control protein E1A	559–975, 1225–1542	559–972, 1224–1541
	Control protein E1A 243 R	559–1113, 1225–1542	559–1110, 1224–1541
E1B	Control protein E1A 19kD	1711–2238	1712–2254
	E1B 55 kDa protein	2016–3503	2017–3516
pIX	Protein IX	3600–4022	3617–4039
pIVa2	Virion morphogenesis protein	(4081–5418, 5697–5708)c	(4098–5435, 5714–5725)c
E2B	E2B DNA polymerase	(5187–8774, 14100–14108)c	(5204–8791, 14116–14124)c
	E2B DNA terminal protein	(8573–10579, 14100–14108)c	(8590–10599, 14116–14124)c
L1	Protein 13.6 K	7968–9657	7985–9677
	L1 52.5 kDa protein	11040–12287	11060–12307
	L1 IIIa protein	12308–14065	12328–14085
L2	L2 Penton base	14145–15869	14161–15885
	L2 VII protein	15876–16472	15892–16488
	L2 V protein	16542–17648	16558–17664
	L2 X protein	17676–17918	17692–17934
L3	L3 VI protein	18001–18749	18017–18769
	L3 hexon protein	18840–21746	18856–21762
	L3 protease	21779–22393	21795–22409
E2A	E2A DNA-binding protein	(22492–24081)c	(22508–24097)c
L4	100 kDa protein	24110–26530	24126–26546
	22 kDa protein	26241–26825	26257–26841
	33 kDa protein	26241–26555, 26758–27126	26257–26572, 26775–27142
	VIII protein	27214–27897	27230–27913
E3	E3 12.5-kDa protein	27898–28221	27914–28237
	E3 CR1-α	28626–28811	28642–28827
	E3 Glycoprotein	28808–29287	28824–29303
	E3 CR1-β	29464–29769	29480–29785
	E3 RID-α	29777–30052	29793–30068
	E3 RID-β	30055–30447	30071–30463
	E3 14.7-kDa protein	30440–30826	30456–30842
U exon	U protein	(30852–31015)c	(30868–31031)c
L5	Fiber	31026–32774	31042–32790
E4	E4 ORF6/7 protein	(32910–33188, 33900–34073)c	(32928–33206, 33915–34089)c
	E4 34-kDa protein	(33189–34073)c	(33205–34089)c
	E4 ORF4 protein	(33994–34338)c	(34010–34354)c
	E4 ORF3 protein	(34350–34700)c	(34366–34716)c
	E4 ORF2 protein	(34697–35089)c	(34713–35105)c
	E4 ORF1 protein	(35139–35525)c	(35156–35542)c

Note: A total of 38 putative coding regions for both Shanxi HAdV strains were identified.

Results of sequence analysis confirmed the high sequence identity between the genomes of the two Shanxi strains. Pairwise alignment using MEGA and Sequencher software revealed a high sequence similarity of 99.6% between two strains, with 134 nucleotide variations, including 105 base substitutions and 29 indels (five single-base indels, one two-base indel, one three-base indel, one four-base indel, and one 15-base indel). Comparative analysis with the six HAdV-C prototype strains, namely, HAdV-1, 2, 5, 6, 57, and 89, showed that strains SX-2000-140 and SX-2004-327 had the highest sequence identities of 98.8% and 99.1% with the HAdV-2 prototype strain (NC_001405-USA-1953), respectively. Furthermore, the two strains showed the lowest sequence identities of 93.5% and 93.6% with the HAdV-5 prototype strain (AC_000008-5-USA-1953), respectively. The genomes of the Shanxi strains showed a high degree of conservation with those of the HAdV-C prototype strains. The highest genetic diversity was found in the sequences of the hexon gene (nt: 1.3–18.4%; aa: 0–15.9%) and fiber gene (nt: 0.3–31.3%; aa: 0.4–32.2%).

Comparative analysis with the other 27 available HAdV-C WGSs indicated that the two Shanxi strains were most closely related to another two Chinese strains, namely, KR699642-2009-CHN (strain CBJ113, isolated from a patient with severe acute respiratory infection in Beijing of China in 2009) (nucleotide similarity 99.5%/99.7%) and MF315029-2013-CHN (strain BJ09, isolated from a patient with respiratory infection in Beijing of China in 2013) (nucleotide similarity 99.4%/99.4%). The average differences in the coding regions of a nonstructural protein (DNA polymerase, E2A-DBP, and L4-100kDa), major capsid proteins (penton base, hexon, and fiber), minor capsid proteins (L1-pIIIa and L3-pVI), and core proteins (pTP and L2-pVII) ranged from 0.2–1.1% in the nucleotide sequences and 0–0.8% in the amino acid sequences.

### Phylogenetic analysis

**Figure 1:**
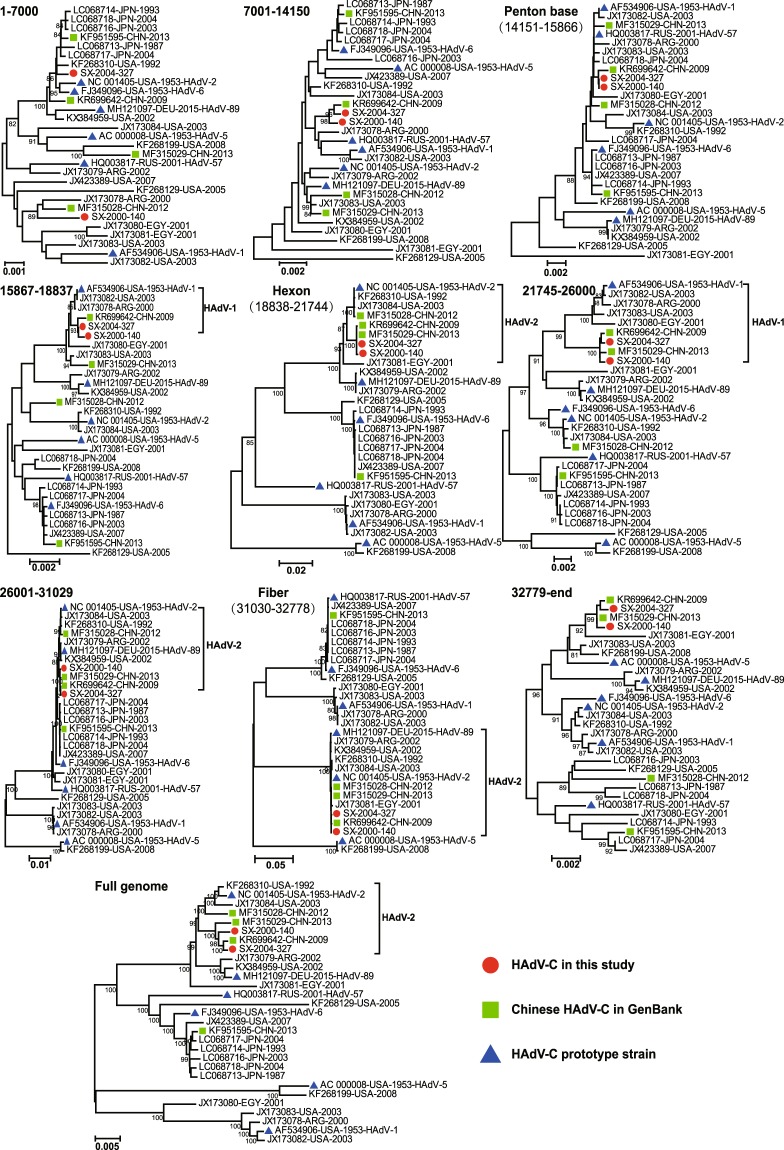
Neighbor-joining phylogenetic tree based on WGS of 29 HAdV-C strains. The nine genomic regions were used to generate the trees based on the reference HAdV-2 prototype strain (GenBank accession number NC_001405). Only bootstrap values greater than 80% are displayed, which indicate the strong support.

**Figure 2:**
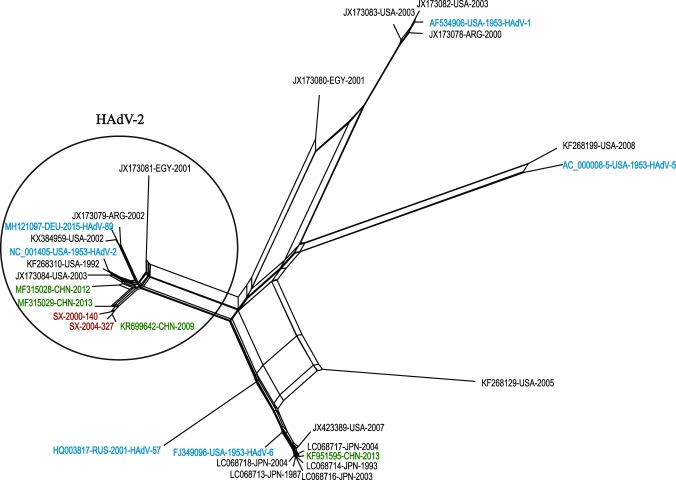
Phylogenetic network generated based on WGS of 29 HAdV-C strains. Red color indicates the two virus strains in this study; green color indicates the Chinese HAdV-C strains downloaded from the GenBank database; blue color indicates the prototype stains of six HAdV-C types, namely, HAdV-1, HAdV-2, HAdV-5, HAdV-6, HAdV-57, and HAdV-89. The fit index for the network was 97.58.

Phylogenetic analysis was further performed to investigate the genetic relationships among the two Shanxi strains and the 27 HAdV-C strains available in the GenBank. Following previously described protocols^20^, WGS was split to nine sequence fragments, namely, nt1-7000, nt7001-14150, penton base gene, nt15867-18837, hexon gene, nt21745-26000, nt26001-31029, fiber gene, nt32779-end (the numbering is based on the NC_001405 genome). A total of ten phylogenetic trees were generated based on WGS and above nine fragments with the neighbor-joining and maximum likelihood method, respectively (Fig. 1 and Supplementary Fig. S1). Both trees showed the consistent results, which revealed a high degree of sequence homology between strains SX-2000-140 and SX-2004-327, and these two Shanxi strains were clustered with the Chinese strain KR699642-CHN-2009 with significant bootstrap support (>95%), except for the trees based on the nt1-7000 region and penton gene. In addition, the two Shanxi strains clustered together with the other Chinese strain MF315029-CHN-2013 in the last six trees based on the hexon gene, nt21745-26000, nt26001-31029, fiber gene, nt32779-end regions and WGS (Fig. 1). Accordingly, the two Shanxi strains are likely to share a common origin with strains KR699642-CHN-2009 and MF315029-CHN-2013. Furthermore, a phylogenetic network was constructed using the 27 HAdV-C WGSs for further evolutionary analysis. The two Shanxi strains, together with the two Chinese strains (KR699642-CHN-2009 and MF315029-CHN-2013), are displayed as parallel lines and can be observed to differ only at the end of a branch (Fig. 2), which further indicated that these strains were closely related and had similar evolutionary histories.

Among the ten phylogenetic trees, the following six trees showed a clear genetic relationship between the two Shanxi strains and the six HAdV prototype strains with strong bootstrap support (Fig. 1): nt15867-18837 (HAdV-1), hexon (HAdV-2), nt21745-26000 (HAdV-1), nt26001-31029 (HAdV-2), fiber (HAdV-2), and WGS (HAdV-2). These results suggested the occurrence of recombination events for the two target Shanxi strains.

### Genetic recombination analysis

**Figure 3:**
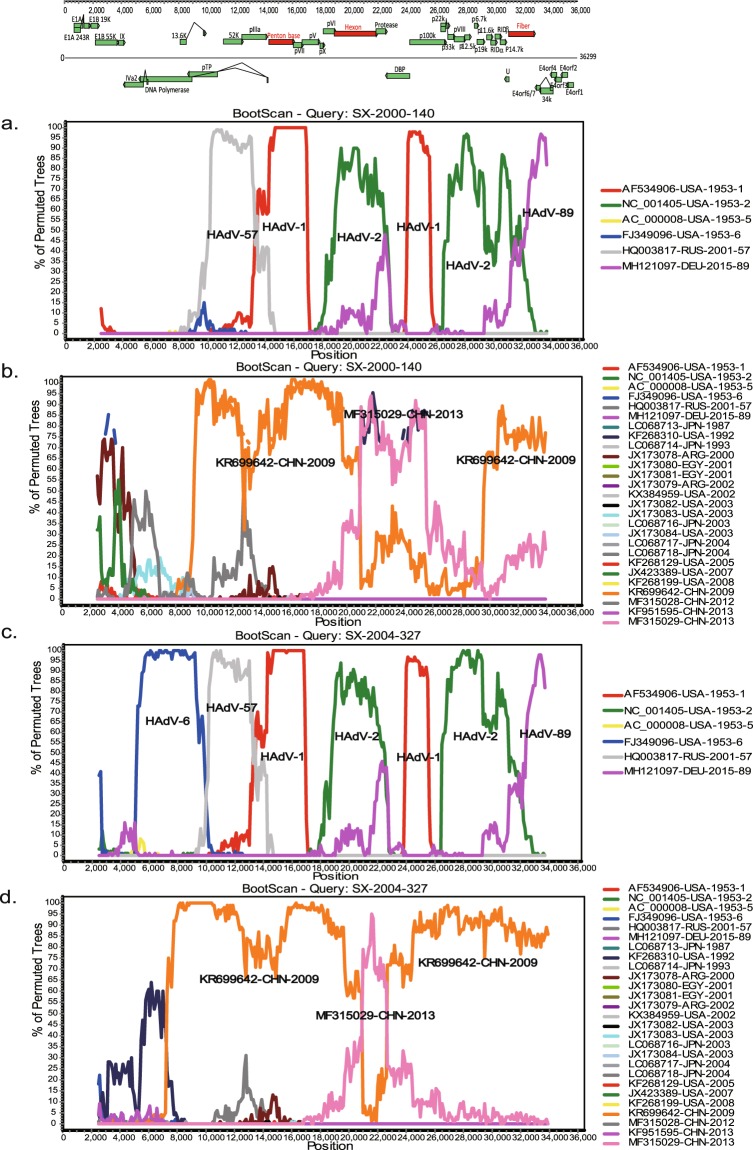
Genetic recombinant analysis of WGS of the two Shanxi strains. The sequences of strains SX-2000-140 (**a**) and SX-2004-327 (**c**) were used as the query sequences against the prototype stains of six HAdV-C types. The sequences of strains SX-2000-140 (**b**) and SX-2004-327 (**d**) were used as the query sequences against the sequences of the four Chinese HAdV-C strains and 23 foreign HAdV-C strains downloaded from the GenBank database. The image shows the organization of the HAdV-C genome. Parameters were set to the default settings as follows: window size of 5000 bp, step size of 100 bp, gap stripping, 100 replicates, kimura (2-parameter), and neighbor-joining.

To investigate the potential recombination events within the genomes of the two Shanxi strains, recombination analyses were performed using SimPlot software (Fig. 3). Both Shanxi strains showed similar recombination patterns with that of the HAdV-C prototype strains, except for the 5ʹ end of the genome. In particular, SX-2000-140 was produced from recombination events among HAdV-57, HAdV-1, HAdV-2, and HAdV-89. In addition, strain SX-2004-327 was generated from recombination events involving HAdV-6, HAdV-57, HAdV-1, HAdV-2, and HAdV-89. The above results indicated the occurrence of multiple intra-typic recombination events. Further analysis with 27 HAdV-C WGSs showed that the genomes of the two Shanxi strains predominantly comprised gene regions derived from KR699642-CHN-2009 and MF315029-CHN-2013, especially the former strain. On the other hand, the 5ʹ ends of the two Shanxi strains (approximately 7000-9000 bp) were highly divergent from those of the other 27 HAdV-C WGSs, which indicated that recombination events within this region likely occurred; however, but the origin might be unknown.

## Discussion

HAdV is responsible for about 5% to 10% of acute respiratory infections in pediatric patients less than five years old worldwide^23^. In particular, HAdV-C viruses are the most prevalent^24,25^. Homologous recombination plays an important role in the molecular evolution of HAdVs and has been previously confirmed for HAdV-A, HAdV-B, and HAdV-D^26^. However, few studies have investigated HAdV-C. To date, at least three lineages related to recombinant HAdV-C have been identified in China^19,20^. Among these three lineages, Lineage 1, which includes strains KR699642-CHN-2009 and MF315029-CHN-2013, were collected in Beijing in 2009 and 2013, respectively. Lineage 2, which includes strain MF315028-2012-CHN isolated from Beijing in 2012, was related to the HAdV-2 prototype strain (NC_001405-USA-1953). Lineage 3, which includes strain KF951595-CHN-2013 isolated from Liaoning province in 2013, was found to be more closely related to Japan HAdV viruses^20^. In addition, a novel recombinant HAdV-C strain (SH2016) was recently reported from an infant case with severe acute respiratory infection in Shanghai of China^27^. This virus recombined with HAdV-1 and HAdV-2 was different from the viruses mentioned above and belonged to the new recombinant form of HAdV-C^27^. However, this sequence had not yet been released from GenBank database, so it could not be introduced into analysis in this study. In the present study, whole-genome sequencing and bioinformatics analysis of two HAdV-C strains (SX-2000-140 and SX-2004-327) isolated from the fecal samples of healthy children were performed. Results revealed that both Shanxi strains had the highest homology and harbored genomic elements of two Chinese HAdV-C strains (KR699642-CHN-2009 and MF315029-CHN-2013) within Lineage 1, thereby indicating that the two target Shanxi strains belonged to Lineage 1 of HAdV-C and share a common ancestor with strains KR699642-CHN-2009 and MF315029-CHN-2013. Considering that the two target Shanxi strains were collected in 2000 and 2004, respectively, we presumed that Lineage 1 of HAdV-C was the domestic strain circulating in mainland of China for decades. On the other hand, viruses within Lineage 1 showed slightly different recombinant patterns, especially at the 5ʹ ends of their genomes, which could be attributed to the progressive accumulation of natural variations and recombinant events throughout their evolutionary histories. However, the lack of HAdV-C sequences collected over time and from other regions worldwide could limit the ability to fully resolve the evolutionary histories of the different HAdV-C lineages.

Consistent with previously reported findings^26^, our results confirmed the high degree of sequence conservation among the HAdV-C viruses, and the major genetic differences between Lineage 1 and prototype strains were found only in the genes encoding the hexon and fiber. Considering that these two proteins are involved in interaction with cellular receptors and host immune response, extremely high variability at the nucleotide and amino acid levels revealed a high degree of immune pressure^17^. The Shanxi strains and two other Beijing strains within Lineage 1 showed slightly different recombinant patterns resulting from recombination events among the five types of HAdV-C^19,20^. However, Lineage 1 strains shared high sequence identities with the HAdV-2 prototype strain (NC_001405-USA-1953) across the genome, especially in the major capsid genes (hexon and fiber), which suggested that Lineage 1 viruses associated with recombinants shared a common ancestor that is closely related to HAdV-2. Considering those genes mediate the attachment of HAdV to cells, the tropism of Lineage1 viruses should be similar to that of HAdV-2.

Considering the long-term persistent infections caused by HAdV-C viruses^16^, frequent co-infections could provide the opportunity for intratypic homologous recombination and further increase the diversity of genetic recombination patterns. Our current findings confirmed that the frequent recombination events among the HAdV-C types could be a major driving force for the molecular evolution of HAdV-C. With the emergence of the recombinant viral pathogens, original “non-pathogenic” or “low-pathogenic” viruses could give rise to “high-pathogenic” strains and further lead to serious public health concerns. For example, strain KR699642-CHN-2009 within Lineage 1 could lead to severe acute respiratory infection among children^19^. This have been also previously observed in the HAdV-55 epidemic, in which recombination between HAdV-B11 and HAdV-B14 gradually became the major etiological agent for pneumonia infections worldwide since it was discovered in China in 2006^28,29^. Therefore, further comprehensive and systematic monitoring, detection, and research on HAdV-C are highly necessary and worthwhile.

## Supplementary information

Supplementary material
