# Supplementary material for: Human adenovirus species C recombinant virus continuously circulated in China
Source: Sci Rep. 2019 Jul 5;9:9781. doi: 10.1038/s41598-019-46228-2 (PMC6611784; doi:10.1038/s41598-019-46228-2)
Supplement: Supplementary file 1 — Supplementary material [file 41598_2019_46228_MOESM1_ESM.pdf]

Human adenovirus species C recombinant virus continuously circulated in China

Jianfang Yang[1#], Naiying Mao[2#], Chuangye Zhang[1], Binzhi Ren[1], Hong Li [1], Na Li[1], Jing Chen[1], Ruifu Zhang[1], Hong Li[2,3], Zhen Zhu[2*], Wenbo Xu[2*]

Supplementary Table S1. The list of 27 HAdV-C strains worldwide downloaded from GenBank database.

GenBank No.	Strain Name	Country	Year	Prototype
AF534906	/	USA	1953	HAdV-1
NC_001405	/	USA	1953	HAdV-2
AC_000008	/	USA	1953	HAdV-5
FJ349096	Tonsil 99 prototype	USA	1953	HAdV-6
HQ003817	human/RUS/16700/2001/57[P1H57F6]	RUS	2001	HAdV-57
MH121097	29C2	DEU	2015	HAdV-89
LC068713	870550	JPN	1987	/
KF268310	human/USA/Pitts_00109/1992/2[P2H2F2]	USA	1992	/
LC068714	930113	JPN	1993	/
JX173078	human/ARG/A15812/2000/1[P1H1F1]	ARG	2000	/
JX173080	human/EGY/E13/2001/1[P1H1F1]	EGY	2001	/
JX173081	human/EGY/E53/2001/1[P2H2F2]	EGY	2001	/
JX173079	human/ARG/A15932/2002/2[P2H2F2]	ARG	2002	/
KX384959	T215/Ft Jackson South Carolina USA/2002	USA	2002	/
LC068716	1030787	JPN	2003	/
JX173082	human/USA/VT384/2003/1[P1H1F1]	USA	2003	/
JX173083	human/USA/VT2672/2003/1[P1H1F1]	USA	2003	/
JX173084	human/USA/VT5544/2003/2[P2H2F2]	USA	2003	/
LC068717	1040264	JPN	2004	/

LC068718	1040502	JPN	2004	/
KF268129	human/USA/UFL_Adv6/2005/6[P6H6F6]	USA	2005	/
JX423389	human/USA/ak31_Adv6/2007/6[P6H6F6]	USA	2007	/
KF268199	human/USA/UFL_Adv5/2008/5[P2H5F5]	USA	2008	/
KR699642	CBJ113	CHN	2009	/
MF315028	human/CHN/BJ04/2012/[P1H2F2]	CHN	2012	/
MF315029	human/CHN/BJ09/2013/[P1H2F2]	CHN	2013	/
KF951595	DD28	CHN	2013	/

Supplementary Figure S1. Maximum likelihood phylogenetic tree based on WGS of 29 HAdV-C strains. The nine genomic regions were used to generate the trees based on the reference HAdV-2 prototype strain (GenBank accession number NC_001405). Only bootstrap values greater than 80% are displayed.

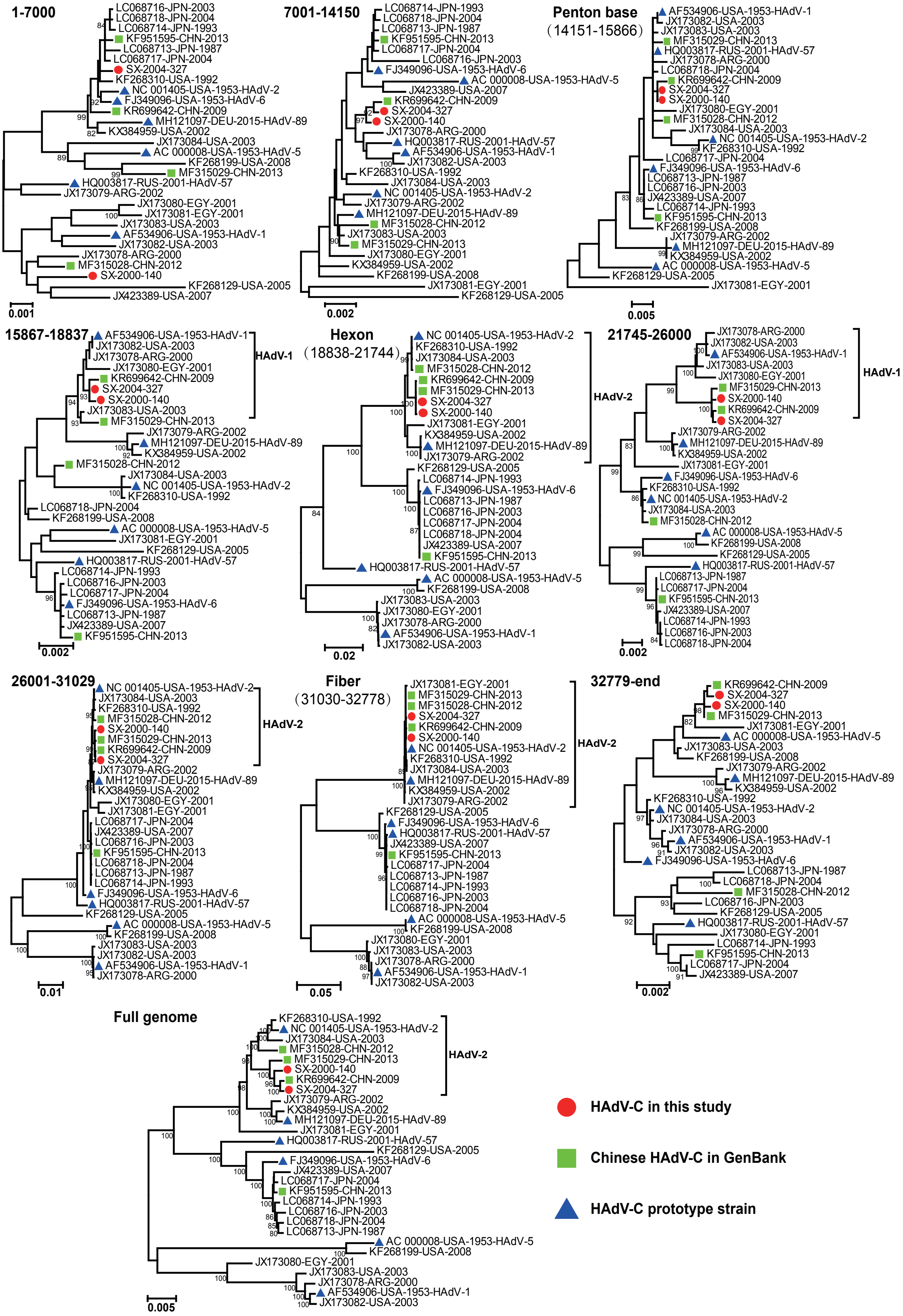
1-7000
LC068716-JPN-2003
LC068718-JPN-2004
LC068714-JPN-1993
KF951595-CHN-2013
LC068713-JPN-1987
LC068717-JPN-2004
SX-2004-327
KF268310-USA-1992
NC 001405-USA-1953-HAdV-2
FJ349096-USA-1953-HAdV-6
KR699642-CHN-2009
MH121097-DEU-2015-HAdV-89
KX384959-USA-2002
JX173084-USA-2003
AC 000008-USA-1953-HAdV-5
KF268199-USA-2008
MF315029-CHN-2013
HQ003817-RUS-2001-HAdV-57
JX173079-ARG-2002
JX173080-EGY-2001
JX173081-EGY-2001
JX173083-USA-2003
AF534906-USA-1953-HAdV-1
JX173082-USA-2003
JX173078-ARG-2000
MF315028-CHN-2012
SX-2000-140
KF268129-USA-2005
JX423389-USA-2007
0.001
7001-14150
LC068714-JPN-1993
LC068718-JPN-2004
LC068713-JPN-1987
KF951595-CHN-2013
LC068717-JPN-2004
LC068716-JPN-2003
FJ349096-USA-1953-HAdV-6
AC 000008-USA-1953-HAdV-5
JX423389-USA-2007
KR699642-CHN-2009
SX-2004-327
SX-2000-140
JX173078-ARG-2000
HQ003817-RUS-2001-HAdV-57
AF534906-USA-1953-HAdV-1
JX173082-USA-2003
KF268310-USA-1992
JX173084-USA-2003
NC 001405-USA-1953-HAdV-2
JX173079-ARG-2002
MH121097-DEU-2015-HAdV-89
MF315028-CHN-2012
JX173083-USA-2003
MF315029-CHN-2013
JX173080-EGY-2001
KX384959-USA-2002
KF268199-USA-2008
JX173081-EGY-2001
KF268129-USA-2005
0.002
Penton base
(14151-15866)
AF534906-USA-1953-HAdV-1
JX173082-USA-2003
JX173083-USA-2003
MF315029-CHN-2013
HQ003817-RUS-2001-HAdV-57
JX173078-ARG-2000
LC068718-JPN-2004
KR699642-CHN-2009
SX-2004-327
SX-2000-140
JX173080-EGY-2001
MF315028-CHN-2012
JX173084-USA-2003
NC 001405-USA-1953-HAdV-2
KF268310-USA-1992
LC068717-JPN-2004
FJ349096-USA-1953-HAdV-6
LC068713-JPN-1987
LC068716-JPN-2003
JX423389-USA-2007
LC068714-JPN-1993
KF951595-CHN-2013
KF268199-USA-2008
JX173079-ARG-2002
MH121097-DEU-2015-HAdV-89
KX384959-USA-2002
AC 000008-USA-1953-HAdV-5
KF268129-USA-2005
JX173081-EGY-2001
0.005
15867-18837
AF534906-USA-1953-HAdV-1
JX173082-USA-2003
JX173078-ARG-2000
JX173080-EGY-2001
KR699642-CHN-2009
SX-2004-327
SX-2000-140
JX173083-USA-2003
MF315029-CHN-2013
HAdV-1
JX173079-ARG-2002
MH121097-DEU-2015-HAdV-89
KX384959-USA-2002
MF315028-CHN-2012
JX173084-USA-2003
NC 001405-USA-1953-HAdV-2
KF268310-USA-1992
LC068718-JPN-2004
KF268199-USA-2008
AC 000008-USA-1953-HAdV-5
JX173081-EGY-2001
KF268129-USA-2005
HQ003817-RUS-2001-HAdV-57
LC068714-JPN-1993
LC068716-JPN-2003
LC068717-JPN-2004
FJ349096-USA-1953-HAdV-6
LC068713-JPN-1987
JX423389-USA-2007
KF951595-CHN-2013
0.002
Hexon
(18838-21744)
NC 001405-USA-1953-HAdV-2
KF268310-USA-1992
JX173084-USA-2003
MF315028-CHN-2012
KR699642-CHN-2009
MF315029-CHN-2013
SX-2004-327
SX-2000-140
JX173081-EGY-2001
KX384959-USA-2002
MH121097-DEU-2015-HAdV-89
JX173079-ARG-2002
HAdV-2
KF268129-USA-2005
LC068714-JPN-1993
FJ349096-USA-1953-HAdV-6
LC068713-JPN-1987
LC068716-JPN-2003
LC068717-JPN-2004
LC068718-JPN-2004
JX423389-USA-2007
KF951595-CHN-2013
HQ003817-RUS-2001-HAdV-57
AC 000008-USA-1953-HAdV-5
KF268199-USA-2008
JX173083-USA-2003
JX173080-EGY-2001
JX173078-ARG-2000
AF534906-USA-1953-HAdV-1
JX173082-USA-2003
0.02
21745-26000
JX173078-ARG-2000
JX173082-USA-2003
AF534906-USA-1953-HAdV-1
JX173083-USA-2003
JX173080-EGY-2001
MF315029-CHN-2013
SX-2000-140
KR699642-CHN-2009
SX-2004-327
HAdV-1
JX173079-ARG-2002
MH121097-DEU-2015-HAdV-89
KX384959-USA-2002
JX173081-EGY-2001
FJ349096-USA-1953-HAdV-6
KF268310-USA-1992
NC 001405-USA-1953-HAdV-2
JX173084-USA-2003
MF315028-CHN-2012
AC 000008-USA-1953-HAdV-5
KF268199-USA-2008
KF268129-USA-2005
HQ003817-RUS-2001-HAdV-57
LC068713-JPN-1987
LC068717-JPN-2004
KF951595-CHN-2013
JX423389-USA-2007
LC068714-JPN-1993
LC068716-JPN-2003
LC068718-JPN-2004
0.002
26001-31029
NC 001405-USA-1953-HAdV-2
JX173084-USA-2003
KF268310-USA-1992
MF315028-CHN-2012
SX-2000-140
MF315029-CHN-2013
KR699642-CHN-2009
SX-2004-327
HAdV-2
JX173079-ARG-2002
MH121097-DEU-2015-HAdV-89
KX384959-USA-2002
JX173080-EGY-2001
JX173081-EGY-2001
LC068717-JPN-2004
JX423389-USA-2007
LC068716-JPN-2003
KF951595-CHN-2013
LC068718-JPN-2004
LC068713-JPN-1987
LC068714-JPN-1993
FJ349096-USA-1953-HAdV-6
HQ003817-RUS-2001-HAdV-57
KF268129-USA-2005
AC 000008-USA-1953-HAdV-5
KF268199-USA-2008
JX173083-USA-2003
JX173082-USA-2003
AF534906-USA-1953-HAdV-1
JX173078-ARG-2000
0.01
Fiber
(31030-32778)
JX173081-EGY-2001
MF315029-CHN-2013
MF315028-CHN-2012
SX-2004-327
KR699642-CHN-2009
SX-2000-140
NC 001405-USA-1953-HAdV-2
KF268310-USA-1992
JX173084-USA-2003
MH121097-DEU-2015-HAdV-89
KX384959-USA-2002
JX173079-ARG-2002
HAdV-2
KF268129-USA-2005
FJ349096-USA-1953-HAdV-6
HQ003817-RUS-2001-HAdV-57
JX423389-USA-2007
KF951595-CHN-2013
LC068717-JPN-2004
LC068713-JPN-1987
LC068714-JPN-1993
LC068716-JPN-2003
LC068718-JPN-2004
AC 000008-USA-1953-HAdV-5
KF268199-USA-2008
JX173080-EGY-2001
JX173083-USA-2003
JX173078-ARG-2000
AF534906-USA-1953-HAdV-1
JX173082-USA-2003
0.05
32779-end
KR699642-CHN-2009
SX-2004-327
SX-2000-140
MF315029-CHN-2013
JX173081-EGY-2001
AC 000008-USA-1953-HAdV-5
JX173083-USA-2003
KF268199-USA-2008
JX173079-ARG-2002
MH121097-DEU-2015-HAdV-89
KX384959-USA-2002
KF268310-USA-1992
NC 001405-USA-1953-HAdV-2
JX173084-USA-2003
JX173078-ARG-2000
AF534906-USA-1953-HAdV-1
JX173082-USA-2003
FJ349096-USA-1953-HAdV-6
LC068713-JPN-1987
LC068718-JPN-2004
MF315028-CHN-2012
LC068716-JPN-2003
KF268129-USA-2005
HQ003817-RUS-2001-HAdV-57
JX173080-EGY-2001
LC068714-JPN-1993
KF951595-CHN-2013
LC068717-JPN-2004
JX423389-USA-2007
0.002
Full genome
KF268310-USA-1992
NC 001405-USA-1953-HAdV-2
JX173084-USA-2003
MF315028-CHN-2012
MF315029-CHN-2013
SX-2000-140
KR699642-CHN-2009
SX-2004-327
HAdV-2
JX173079-ARG-2002
KX384959-USA-2002
MH121097-DEU-2015-HAdV-89
JX173081-EGY-2001
HQ003817-RUS-2001-HAdV-57
KF268129-USA-2005
FJ349096-USA-1953-HAdV-6
JX423389-USA-2007
LC068717-JPN-2004
KF951595-CHN-2013
LC068714-JPN-1993
LC068716-JPN-2003
LC068718-JPN-2004
LC068713-JPN-1987
AC 000008-USA-1953-HAdV-5
KF268199-USA-2008
JX173080-EGY-2001
JX173083-USA-2003
JX173078-ARG-2000
AF534906-USA-1953-HAdV-1
JX173082-USA-2003
0.005
HAdV-C in this study
Chinese HAdV-C in GenBank
HAdV-C prototype strain

Reference

1. Wold WSM IM. Adenoviruses. In: Knipe DM, Howley PM, Cohen JI, Griffin GE, Lamb RA, Martin MA, et al., eidtors. Fields virology. 6th ed. Philadelphia Lippincott Williams&Wilkins, p. 1732-1767 (2013).

2. Davison, A.J., Benko, M. & Harrach, B. Genetic content and evolution of adenoviruses. *J Gen Virol* **84**, 2895-2908, https://doi.org/10.1099/vir.0.19497-0 (2003).

3. Madisch, I., Harste, G., Pommer, H. & Heim, A. Phylogenetic analysis of the main neutralization and hemagglutination determinants of all human adenovirus prototypes as a basis for molecular classification and taxonomy. *J Virol* **79**, 15265-15276, https://doi.org/ 10.1128/JVI.79.24.15265-15276.2005 (2005).

4. Walsh, M.P. *et al*. Computational analysis identifies human adenovirus type 55 as a re-emergent acute respiratory disease pathogen. *J Clin Microbiol* **48**, 991-993, https://doi.org/10.1128/JCM.01694-09 (2010).

5. Chen, M. *et a*l. Adenoviruses associated with acute respiratory diseases reported in Beijing from 2011 to 2013. *PloS one* **10**, e0121375, https://doi.org/ 10.1371/journal.pone.0121375 (2015).

6. Lei, Z. *et al*. Outbreaks of epidemic keratoconjunctivitis caused by human adenovirus type 8 in the Tibet Autonomous Region of China in 2016. *PloS one* **12**, e0185048, https://doi.org/ doi: 10.1371/journal.pone.0185048 (2017).

7. Huang, Y.C. *et al*. Adenovirus infection associated with central nervous system dysfunction in children. *J Clin Virol* **57**, 300-304, https://doi.org/

10.1016/j.jcv.2013.03.017 (2013).

8. Portal, T.M. *et al*. Detection and genotyping of enteric viruses in hospitalized children with acute gastroenteritis in Belem, Brazil: Occurrence of adenovirus viremia by species F, types 40/41. *J Med Virol* ***91***, 378-384, https://doi.org/10.1002/jmv.25321 (2019).

9. Kajon, A.E., Portes, S.A., de Mello, W.A., Nascimento, J.P. & Siqueira, M.M. Genome type analysis of Brazilian adenovirus strains of serotypes 1,2,3,5, and 7 collected between 1976 and 1995. *J Med Virol* **58**, 408-412 (1999).

10. Scott, M.K. *et al*. Human Adenovirus Associated with Severe Respiratory Infection, Oregon, USA, 2013-2014. *Emerg Infect Dis* **22**, 1044-1051, https://doi.org/10.3201/eid2206.151898 (2016).

11. Thounaojam, A.D., Balakrishnan, A. & Mun, A.B. Detection and Molecular Typing of Human Adenoviruses Associated with Respiratory Illnesses in Kerala. *Jpn J Infect Dis* **69**, 500-504, https://doi.org/10.7883/yoken.JJID.2015.414 (2016).

12. Edwards, K.M., Thompson, J., Paolini, J. & Wright, P.F. Adenovirus infections in young children. *Pediatrics* **76**, 420-424 (1985).

13. Tebruegge, M. & Curtis N. Adenovirus: an overview for pediatric infectious diseases specialists. *Pediatr Infect Dis* J **31**, 626-627, https://doi.org/10.1097/INF.0b013e318250b066 (2012).

14. Ison, M.G. Adenovirus infections in transplant recipients. *Clin Infect Dis* **43**, 331-339, https://doi.org/10.1086/505498 (2006).

15. Cassir, N. *et al*. Outbreak of adenovirus type 1 severe pneumonia in a French

intensive care unit, September-October 2012. *Euro Surveill* **19**, pii: 20914 (2014).

16. Garnett, C.T. *et al*. Latent species C adenoviruses in human tonsil tissues. *J Virol* **83**, 2417-2428, https://doi.org/10.1128/JVI.02392-08 (2009).

17. Walsh, M.P. *et al*. Computational analysis of two species C human adenoviruses provides evidence of a novel virus. *J Clin Microbiol* **49**, 3482-3490, https://doi.org/10.1128/JCM.00156-11 (2011).

18. Dhingra A. *et al*. Molecular evolution of human adenovirus (HAdVspecies C. *Sci Rep* **9**, 1039, https://doi.org/10.1038/s41598-018-37249-4 (2019).

19. Wang, Y. *et al*. Phylogenetic evidence for intratypic recombinant events in a novel human adenovirus C that causes severe acute respiratory infection in children. *Sci Rep* **6**, 23014, https://doi.org/ 10.1038/srep23014 (2016).

20. Mao, N. *et al*. Whole genomic analysis of two potential recombinant strains within Human mastadenovirus species C previously found in Beijing, China. *Sci Rep*. **7**, 15380, https://doi.org/10.1038/s41598-017-15336-2 (2017).

21. World Health Organization. Isolation and identification of polioviruses. WHO Polio laboratory manual, 4th edn. (2004).

22. Tamura, K. *et al*. MEGA5: molecular evolutionary genetics analysis using maximum likelihood, evolutionary distance, and maximum parsimony methods. *Mol Biol Evol* **28**, 2731-2739, https://doi.org/10.1093/molbev/msr121 (2011).

23. Pavia, A.T. Viral infections of the lower respiratory tract: old viruses, new viruses, and the role of diagnosis. *Clin Infect Dis* **52**, S284-289, https://doi.org/10.1093/cid/cir043 (2011).

24. Mizuta, K. *et al*. Stability of the seven hexon hypervariable region sequences of adenovirus types 1-6 isolated in Yamagata, Japan between 1988 and 2007. *Virus Res* **140**, 32-39, https://doi.org/10.1016/j.virusres.2008.10.014 (2009).

25. Ma, G. *et al*. Species C is Predominant in Chinese Children with Acute Respiratory Adenovirus Infection. *Pediatr Infect Dis J* **34**, 1042, https://doi.org/10.1097/INF.0000000000000791 (2015).

26. Ismail, A.M. *et al*. Genomic analysis of a large set of currently-and historically-important human adenovirus pathogens. *Emerg Microbes Infect* **7**, 10, https://doi.org/10.1038/s41426-017-0004-y (2018).

27. Zhang W & Huang L. Genome Analysis of A Novel Recombinant Human Adenovirus Type 1 in China. *Sci Rep* **9**, 4298, http://doi.rog/10.1038/s41598-018-37756-4 (2019).

28. Zhu, Z. *et al*. Outbreak of acute respiratory disease in China caused by B2 species of adenovirus type 11. *J Clin Microbiol* **47**, 697-703, https://doi.org/10.1128/JCM.01769-08 (2009).

29. Cao, B. *et al*. Emergence of community-acquired adenovirus type 55 as a cause of community-onset pneumonia. *Chest* **145**, 79-86, https://doi.org/10.1378/chest.13-1186 (2014).
